# Dietary recommendations for people with cystic fibrosis, a relevantly changing field

**DOI:** 10.1016/j.jped.2026.101500

**Published:** 2026-01-30

**Authors:** Jochen G. Mainz, Carlos Zagoya

**Affiliations:** a CF-Center, Klinikum Westbrandenburg, Campus Brandenburg an der Havel, Germany; b Medizinische Hochschule Brandenburg (MHB), Universitätsklinikum, Brandenburg an der Havel, Germany

Malnutrition in people with cystic fibrosis was recognized as a generalized problem in 1938, when Dorothy Andersen identified “Cystic fibrosis (CF) of the pancreas” as a feature of the multi-organ inherited disease [1]. Accordingly, in those years, failure to thrive was a principal reason for early death in children with CF. As known today, about 85 % of people with CF (pwCF) reveal early exocrine pancreatic insufficiency (EPI), whereas the remaining 15 % do not reveal early EPI and, consequently, their stable nutritional status mostly does not accord to the phenotype anticipated for pwCF.

As for maldigestion in pwCF with EPI, fat diets had previously been identified as a cause of abundant diarrhea. Consequently, until some 35 years ago, low-fat high-carbohydrate diets were commonly indicated for patients with CF-related EPI. This paradigm was widely applied until 1988, when Corey et al. published “A comparison of survival, growth, and pulmonary function in patients with cystic fibrosis in Boston and Toronto” [2]. In that study, the cystic fibrosis populations in both clinics were highly comparable in regard to age, sex and even population size (Boston: n = 499, mean ± SD age 15.9 ± 9.6, range 0-45 years; Toronto: n = 534, mean ± SD age 15.2 ± 8.3, range 0-43 years) [2]. However, patients in Toronto turned out to be taller, and, despite revealing comparable pulmonary function, median survival in Boston was 21 years, compared to 30 years in Toronto.

The origin of the highly intriguing difference happened to be nutritional guidance and interventions. The low-fat, high-carbohydrate diet had been abandoned in Toronto in favor of enhanced fat intake together with higher dosages in pancreatic enzyme replacement therapies (PERT). As a matter of fact, this resulted in enhanced growth and, ultimately, in a nine year survival advantage compared to pwCF attended in Boston [3]. Finally, this comparative study convinced the worldwide CF communities to supply pwCF with fat enriched meals and higher PERT dosages, as a CF-specific nutritional guideline.

Over time, failure to thrive was eventually replaced by respiratory failure, as the principal reason for premature death of pwCF and different studies proved that severity of malnutrition correlates with the loss of pulmonary function [[4], [5], [6]]. Interestingly, this also manifested in a switch in the specialty fields of directors of CF-centers worldwide. In former times, when severe malnutrition was a leading early symptom, among critical GI courses observed in the 10-20% of pwCF born with meconium-ileus and those later suffering from distal intestinal obstruction syndromes (DIOS), intussusception, prolapses and constipation [3,[7], [8], [9]], it often was pediatric gastroenterologists who led CF centers.

Further improvement in the survival of pwCF was achieved with the establishment of multidisciplinary CF-specialized teams complementing CF centers, whose directors are now predominantly pediatric- and, more recently, also adult pneumologists. In addition to CF-specialized nutritionists, radiologists, diabetologists as well as CF-specialized physiotherapists and psychosocial specialists became an essential part of certified interdisciplinary CF centers.

With that and intensive therapy programs including mobilization of viscous secretions from the airways by inhalation, physiotherapy and sport, as well as high amounts of antibiotics targeting pathogenic bacteria like Pseudomonas aeruginosa colonizing obstructed airways, an increasing proportion of CF patients started to reach adulthood.

Nevertheless, nutritional support became a principal column of therapy, in addition to PERT, including its optimal dosage and timing strategies, nutritional supplementation focusing on high-calory fat-enriched meals and supplementation with fat-soluble vitamins AEDK.

In those times, when life expectancy of pwCF remained relevantly limited, we did not take a major focus on the quality of nutrients. Instead, we focused on high-energy intake. Consequently, frequent fast food intake was not been considered a problem, but rather an opportunity to obtain high amounts of energy with low volumes of food.

The present study on “Diet quality in Brazilian adolescents with cystic fibrosis” by Pascon et al. [10] included 47 children with CF aged 12-19 years. Conducted before highly effective CFTR-modulating therapies (HEMTs) were available, this study underlines the relation between high-quality, non-ultra-processed foods (UPFs) and a better pulmonary function in pwCF.

With the relatively recent availability of HEMTs [11], which in vitro were found to restore CFTR channel’s function to almost 50% of the normal level, the aspect of diet quality is increasingly gaining significant relevance. Targeting the underlying CFTR protein defect, HEMTs refer to ivacaftor alone, predominantly for pwCF carrying a rare gating mutation as HEMTs, as well as elexacaftor/tezacaftor/ivacaftor (ETI) for patients carrying frequent CFTR mutations like F508del. In some countries, the latter group may now switch to the newest triple HEMT, a combination of vanzacaftor/tezacaftor/deutivacaftor.

This relatively wide availability of HEMTs has led to a substantial stabilization of body weight in a large proportion of pwCF [12]. Moreover, and most pronounced in North America, the increase in body weight of patients who previously suffered from malnutrition is now turning into overweight and obesity, a recent problem emerging in CF communities of high-income countries. Accordingly, recent analyses from the US cystic fibrosis foundation patient registry revealed a 40% decrease in underweight in pwCF, together with a > 300% increase in overweight and > 400% increase in obesity between 2000 and 2019 [13,14]. Overweight in adults is defined as a BMI of 25-29.9 kg/m^2^, while obesity is classified as a BMI of 30 kg/m^2^ or higher. For children aged 2 or older, overweight is defined as a BMI between the 85th and the 94.9th percentile or, equivalently, a BMI >1 SD (∼84th percentile) above the WHO growth reference median. Childhood obesity, on the other hand, refers to a BMI ≥ 95th percentile or, equivalently, a BMI z-score >2 SD (∼98th percentile) above the WHO growth reference median [11].

These critical dynamics in CF populations are mainly caused by a combination of factors such as nutritional habits, diet quality and HEMT effects, since HEMT may restore residual exocrine pancreatic function to some variable extent. HEMT were also found to reduce intestinal viscous mucus, which previously impaired resorption of nutrients, and to correct gastric hyperacidity, improving effectiveness of PERT and reduce energy wasting by infection and inflammation.

Nevertheless, chronic inflammation is still a hallmark of CF, albeit to a much lesser extent in pwCF treated with HEMTs [15], and it was found to be present in pwCF, even before airway colonization with pathogens. Together with frequent CF-related diabetes, found in up to 50% of pwCF reaching ages above 40 years [16], overweight and obesity in pwCF will increasingly contribute to the risk of metabolic syndrome in the cohort of patients increasingly reaching older ages [14]. In the end, obesity and high blood pressure may complement risks of metabolic syndromes for cardiovascular pathologies in the aging pwCF [14].

Most interestingly, in a Brazilian cohort of juvenile pwCF not yet receiving HEMT, Pascon et al. [10] found lower consumption of fruit and vegetables together with higher fast-food intake to be associated with more severe lung function loss. At the same time, better BMI/age Z-scores were observed in pwCF who more frequently consumed fruit and vegetables, white meat, and oil. In contrast, frequent highly processed fast food in those who did not have what we often call a “Mediterranean diet” was related to impaired thriving.

Already before HEMT introduction, Pascon et al. [10] emphasize the need for change in current paradigms followed in nutritional therapy for pwCF, which turns out to be mandatory for those newly receiving HEMT. Recommendations include a reduction in fat and fast food consumption, together with an increase in vegetables, fruits, white meat and poly-unsaturated oils, a Mediterranean diet, in accordance with general population guidelines.

Therewith, optimal effects of CFTR-modulating therapies may be achieved, improving pulmonary function and stabilizing the previously often observed reduced body weight without leading to obesity and long term complications contributing to higher risks of the metabolic syndrome. Appropriate dietary counselling should be provided prior to starting CFTR modulators, including discussions about possible weight gain and overweight.

## Conflicts of interest

The authors declare no conflicts of interest.
